# The effect of lengthening contractions on neuromuscular junction structure in adult and old mice

**DOI:** 10.1007/s11357-016-9937-7

**Published:** 2016-07-29

**Authors:** Aphrodite Vasilaki, Natalie Pollock, Ifigeneia Giakoumaki, Katarzyna Goljanek-Whysall, Giorgos K. Sakellariou, Timothy Pearson, Anna Kayani, Malcolm J. Jackson, Anne McArdle

**Affiliations:** MRC-Arthritis Research UK Centre for Integrated Research into Musculoskeletal Ageing, Department of Musculoskeletal Biology, Institute of Ageing and Chronic Disease, University of Liverpool, Liverpool, L7 8TX UK

**Keywords:** Skeletal muscle, Ageing, Lengthening contractions, Regeneration, Innervation neuromuscular junction

## Abstract

Skeletal muscles of old mice demonstrate a profound inability to regenerate fully following damage. Such a failure could be catastrophic to older individuals where muscle loss is already evident. Degeneration and regeneration of muscle fibres following contraction-induced injury in adult and old mice are well characterised, but little is known about the accompanying changes in motor neurons and neuromuscular junctions (NMJs) following this form of injury although defective re-innervation of muscle following contraction-induced damage has been proposed to play a role in sarcopenia. This study visualised and quantified structural changes to motor neurons and NMJs in *Extensor digitorum longus* (EDL) muscles of adult and old *Thy1-YFP* transgenic mice during regeneration following contraction-induced muscle damage. Data demonstrated that the damaging contraction protocol resulted in substantial initial disruption to NMJs in muscles of adult mice, which was reversed entirely within 28 days following damage. In contrast, in quiescent muscles of old mice, ∼15 % of muscle fibres were denervated and ∼80 % of NMJs showed disruption. This proportion of denervated and partially denervated fibres remained unchanged following recovery from contraction-induced damage in muscles of old mice although ∼25 % of muscle fibres were completely lost by 28 days post-contractions. Thus, in old mice, the failure to restore full muscle force generation that occurs following damage does not appear to be due to any further deficit in the percentage of disrupted NMJs, but appears to be due, at least in part, to the complete loss of muscle fibres following damage.

## Introduction

During ageing, skeletal muscles become smaller and weaker such that, in humans, by age 70, the cross-sectional area of skeletal muscle is reduced by 25–30 % and muscle strength is reduced by 30–40 % (Porter et al. 1995). The changes in muscles during ageing show considerable similarities between man and rodents (Garcia et al. 2013; Miller 2004). Thus, most of the intrinsic and extrinsic changes regulating muscle ageing in humans have also been observed in rodents, indicating that mice and rats are good models of sarcopenia in humans (Demontis et al. 2013). Humans, rats and mice show loss of muscle fibres with ageing. The maximum isometric force decreases more than muscle mass during ageing in mice in a similar manner to humans, even when expressed relative to the cross-sectional area of the muscle (Faulkner et al. 1995; Gonzalez and Delbono 2001), demonstrating a weakening of remaining muscle fibres. Denervation is proposed to contribute to loss of muscle mass and function in humans and rodents (Delbono 2003; Jang and Van Remmen 2011), and loss of fibres is associated with a loss of motor units in humans, rats and mice (Brown et al. 1988; Campbell et al. 1973; Einsiedel and Luff 1992; Larsson and Ansved 1995; Lexell et al. 1986, 1988). The muscle fibres that remain show an increased susceptibility to damage and a substantially reduced ability to fully regenerate following damage (Brooks and Faulkner 1990; Kayani et al. 2008; McArdle et al. 2004), potentially resulting in a permanent functional deficit when damage occurs. This failure of muscle from older individuals to regenerate fully following damage could be catastrophic to older individuals. It has also been proposed that multiple damaging insults may be a key mechanism leading to loss of muscle fibres during ageing (Ehrhardt and Morgan 2005; Shi and Garry 2006), a process which contributes substantially to sarcopenia, although the role of failed regeneration in this process is controversial since Fry et al. have shown that although depleting muscle satellite cells has a severe effect on regenerative capacity of muscle, this has little effect on sarcopenia (Fry et al. 2015).

The mechanisms by which muscle regeneration following contractile activity fails in old mammals are poorly understood. Studies to date have focussed primarily on the ability of muscles of old rodents to regenerate following treatment with myotoxins, following nerve crush or resection or following grafting of muscles across different aged rodents which initiates spontaneous muscle degeneration together with nerve resection (Carlson et al. 2001; Kawabuchi et al. 2011; Lee et al. 2013; Streppel et al. 1998; Verdu et al. 1995). Degeneration and regeneration of muscle fibres following contraction-induced injury in adult and old mice are well characterised, but little is known about the accompanying changes in motor neurons and neuromuscular junctions following this form of injury. Lengthening contractions cause considerably more damage to skeletal muscle than shortening or isometric contractions (Faulkner et al. 1989; McCully and Faulkner 1985), and the pattern of damage leading to loss of muscle force generation and recovery follows a characteristic pattern and time course (Brooks and Faulkner 1990; Faulkner et al. 1989; McArdle et al. 2004; McCully and Faulkner 1985, 1986). The time course and characteristics of contraction-induced muscle injury and recovery are similar for mice (Brooks et al. 1995; Faulkner et al. 1989) and humans (Faulkner et al. 1993; Friden et al. 1983). Studies in young/adult mice show that the maximum force deficit occurs at approximately 3 days following a period of severe lengthening contractions with subsequent recovery/regeneration resulting in a complete restoration of maximum force generation by 28 days following this damage (Brooks and Faulkner 1990; McArdle et al. 2004). In old mice, the time course of functional recovery following contraction-induced damage is well characterised. Recovery of force generation for up to ∼14 days post-damage appears equivalent to that seen in adult mice (McArdle et al. 2004). In contrast, later recovery appears to fail, and muscles show a significant residual force deficit at 28 days following damaging lengthening contractions that appears permanent (Brooks and Faulkner 1990; McArdle et al. 2004). The cause of this force deficit in muscles of old mice is unclear, but the relatively normal initial phase of regeneration suggests that this may not be related to a deficit in satellite cell activation previously reported in muscle of old mice (Ehrhardt and Morgan 2005; Shi and Garry 2006).

Considerable changes in muscle innervation occur during ageing (Chai et al. 2011; Jang and Van Remmen 2011; Valdez et al. 2010), and there is increasing evidence that the loss of skeletal muscle fibres with ageing is associated with a loss of whole motor units (Brown et al. 1988; Einsiedel and Luff 1992; Larsson and Ansved 1995) although it is unclear whether motor neuron defects are a cause or effect of the loss of muscle fibres (Larsson and Ansved 1995; Wang et al. 2005). Some data from rodents also indicate that, despite the loss of innervation of neuromuscular junctions that occurs with ageing, the number of motor neuron cell bodies in the lumbar spinal cord are unchanged indicating that changes predominantly occur in peripheral regions of motor units (Chai et al. 2011). The mechanisms by which this full or partial denervation of neuromuscular junctions occurs during ageing are unknown, but it has been proposed that the muscle damage that occurs during everyday muscle activities contributes to these changes in innervation.

This study has used a well-established rodent model of muscle damage to test the hypotheses that

(i) lengthening contraction-induced damage to muscles is associated with disruption of the innervation of muscle fibres that would be fully reversed during regeneration in adult mice, but that
(ii) the late stage failure in ability of muscles from old mice to recover their ability to generate force following lengthening contractions is associated with a failure to appropriately re-innervate regenerated muscle fibres.

## Methods

### Ethical approval

Experiments were performed in accordance with UK Home Office guidelines under the UK Animals (Scientific Procedures) Act 1986 and received ethical approval from the University of Liverpool Animal Welfare and Ethical Review Body (AWERB).

### Mice

This study used adult (6–8 months) and old (26–28 months) male Thy1-YFP transgenic mice. Breeding pairs were originally purchased from The Jackson Laboratory (stock number 003709; http://jaxmice.jax.org/strain/003709.html). These transgenic mice express yellow fluorescent protein in high levels in motor and sensory neurons, as well as in subsets of central neurons. This strain provides a strong and specific marker of axons with 100 % YFP expression in motor neurons from mid-gestational stages and allows detailed visualisation of skeletal muscle innervation using confocal microscopy without the use of antibody staining. Mice were bred from homozygous breeding pairs and were fed a standard laboratory diet. Mice were exposed to a 12-h dark, 12-h light cycle.

### Lengthening contraction protocol

Extensor digitorum longus (EDL) muscles were subjected to a well-characterised protocol of 450 lengthening contractions as described previously (McArdle et al. 2004; McCully and Faulkner 1985). Briefly, adult and old mice were anaesthetised using gas anaesthesia (2 % isoflurane in 2 l/min oxygen delivered via a precision vaporizer). The knee of the right hind-limb was fixed. The distal tendon was exposed and attached to the lever arm of a servomotor (Cambridge Technology, UK). The lever served as a force and displacement transducer. The peroneal nerve was exposed, and electrodes were placed across the nerve. The EDL muscle of the contralateral limb served as a control. The protocol of 450 lengthening contractions consisted of three 5-min periods with 150 nerve-induced contractions during each period (i.e. one contraction every 2 s) and a 5-min rest period between each lengthening contraction period. This protocol produces an equivalent level of damage to 60–70 % of the muscle fibres in muscles from adult and old mice allowing the study of regeneration following an equivalent level of damage in muscles of adult and old mice (McArdle et al. 2004; Rader and Faulkner 2006). The protocol follows a well-established and characteristic pattern and time course of loss of force generation, followed by regeneration and recovery of force generation by the skeletal muscle that is complete by 28 days in muscles of adult mice, but which fails after 14 days in muscles of old mice (Brooks and Faulkner 1990; Kayani et al. 2008; McArdle et al. 2004). Contractile forces were not measured in this cohort of mice to avoid potential artefactual changes in NMJ structure following exposure of the NMJs to a series of high-frequency contractions. Sham-operated controls were used. The EDL is composed of 99 % fast myofibres, and the focus was on this muscle since it has been shown that fast muscle fibres are preferentially lost in old age. Therefore, fibre-typing was not considered as necessary in our study.

At 3 and 28 days following the contraction protocol, mice were killed by cervical dislocation, and the EDL muscles were immediately removed. EDL muscles from adult and old mice were either mounted directly on a cork disk, surrounded with O.C.T. mounting medium (Fisher Scientific Ltd, UK), frozen rapidly in isopentane cooled in liquid nitrogen and sectioned using a cryostat for histological analysis (tranverse sections) or were fixed in 4 % p-formaldehyde for 30 min prior to sectioning (longitudinal sections).

### Visualisation of NMJs

**Fig. 1:**
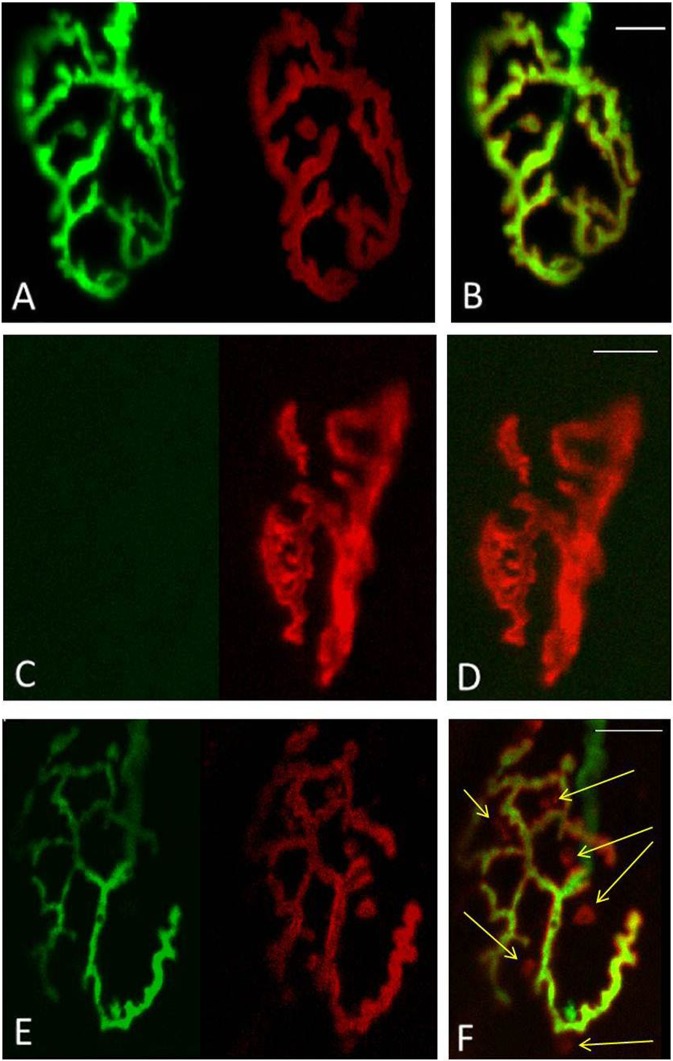
Classification of NMJs. **a–b** fully innervated, **c–d** fully denervated, **e–f** partly innervated with AChR not covered by the nerve terminals identified by *yellow arrow*. **a, c, e** YFP in motor axons (green), AChRs labelled with Alexa-594-α-bungorotoxin (*red*). **b, d, f** Merged image from **a, c, e**. *Scale bar* 10 μm

To visualise AChRs in Thy1-YFP mice, 35-μm longitudinal sections from the EDL muscles were incubated for 30 min with 5μg/ml Alexa 594-conjugated BTX (Molecular Probes, Life Technologies Ltd, UK) as previously described (Valdez et al. 2010). Fluorescence images were obtained using a C1 confocal laser-scanning microscope (Nikon Instruments Europe BV, Surrey, UK) equipped with a 405-nm excitation diode laser, a 488-nm excitation argon laser, and a 543-nm excitation helium-neon laser. Emission fluorescence was detected through a set of 450/35, 515/30 and 605/15 emission filters. Fluorescence images were captured and analysed with the EZC1 V.3.9 (12bit) acquisition software. NMJs that had both green (YFP) and red (BTX) overlapping staining were considered as innervated (Fig. 1a, b), while NMJs stained with only BTX of the postsynaptic AChR site were considered as fully denervated (Fig. 1c, d). Faintly labelled patches of receptors and receptors that were only partially covered by the nerve terminal were considered as fragmented (Fig. 1e, f, marked by yellow arrow). Data was collected from 100 NMJs per EDL from 4 animals at each time point. Red arrows in Figs. 4, 6 and 7 indicate structural changes including nerve terminal fragmentation and partially or fully denervated NMJs.

### Histological analysis of EDL muscles

Transverse sections of muscles (10 μm) were stained with hematoxylin and eosin, dehydrated and mounted in DPX mountant (VWR International Ltd., Poole, UK). Muscles were examined at 3 and 28 days following the lengthening contraction protocol. Images were captured using a Zeiss Axiovert 200M microscope equipped with ×10 and ×20 objectives and analysed using Axiovision 4.4 image capture and analysis software (Carl Zeiss GmbH, Germany).

For Neural Cell Adhesion Molecule (NCAM) immunostaining, transverse sections of muscles (12 μm) were fixed with ice-cold methanol for 10 min, rinsed three times with phosphate buffer saline (PBS) and blocked for 30 min with PBS-0.05 % Tween 20 containing 20 % calf serum (PBST-S) at room temperature as previously described (Kostrominova 2010). Sections were incubated overnight with rabbit anti-neural cell adhesion molecule antibody (NCAM; Chemicon, Temecula, CA, USA) at 4 °C in PBST-S. Sections were washed in PBST-S and were incubated with Alexa Fluor® 532 Goat Anti-Rabbit IgG (Molecular Probes) for 1 h at room temperature. Co-staining of sections with fluorescein-conjugated wheat germ agglutinin (green, WGA–fluorescein, 2 μg/ml; Vector Laboratories, UK) was used for visualisation of the connective tissue around muscle fibres as previously described (Kostrominova 2011). Sections were covered using Vectashield with DAPI (Vector Laboratories, UK) and were visualised using a C1 confocal laser-scanning microscope (Nikon Instruments Europe BV, Surrey, UK). Sections incubated only with Alexa Fluor® 532 Goat Anti-Rabbit IgG were used as negative controls.

### Real-time PCR

RNA isolation and quantitative real-time PCR were performed using standard methods (Goljanek-Whysall et al. 2014). cDNA synthesis (mRNA) was performed using 500 ng RNA and SuperScript II according to the manufacturer’s protocol. qPCR analysis was performed using sso-Advanced SybrGreen Mastermix (Biorad) in a 10-μl reaction as described previously (Soriano-Arroquia et al. 2016). Expression relative to 18S was calculated using delta delta Ct method. The sequences of the primers are shown in Table 1.

**Table 1:** The sequences of the primers used for quantitative real-time PCR

Gene	Forward primer sequence	Reverse primer sequence
18S	GGGGAGTATGGTTGCAAAGC	CGCTCCACCAACTAAGAACG
Atrogin-1	GCAGAGAGTCGGCAAGTC	CAGGTCGGTGATCGTGAG
MuRF1	AGTGTCCATGTCTGGAGGTCGTTT	ACTGGAGCACTCCTGCTTGTAGAT
AchRα (Punga et al. 2011)	GCCATTAACCCGGAAAGTGAC	CCCCGCTCTCCATGAAGTT
MuSK (Punga et al. 2011)	GCCTTCAGCGGGACTGAG	GAGGCGTGGTGACAGG

### Statistics

Statistical analysis of data was undertaken using the Student’s *t* test, and analysis of more than two conditions was performed by ANOVA, using SPSS software. The mouse numbers to produce appropriate sample sizes have been calculated using power calculations based on the likely magnitude of effect from published data and data provided from the author’s laboratories. They are based on changes in each index at conventional 5 % two-sided significance and 80 % power. To obtain this, power required four mice per time point. For real-time PCR experiments, data distribution was assessed using the Mann-Whitney test, and *p* value was calculated using unpaired Student’s *t* test.

## Results

### Histological analysis of EDL muscles following lengthening contractions

**Fig. 2:**
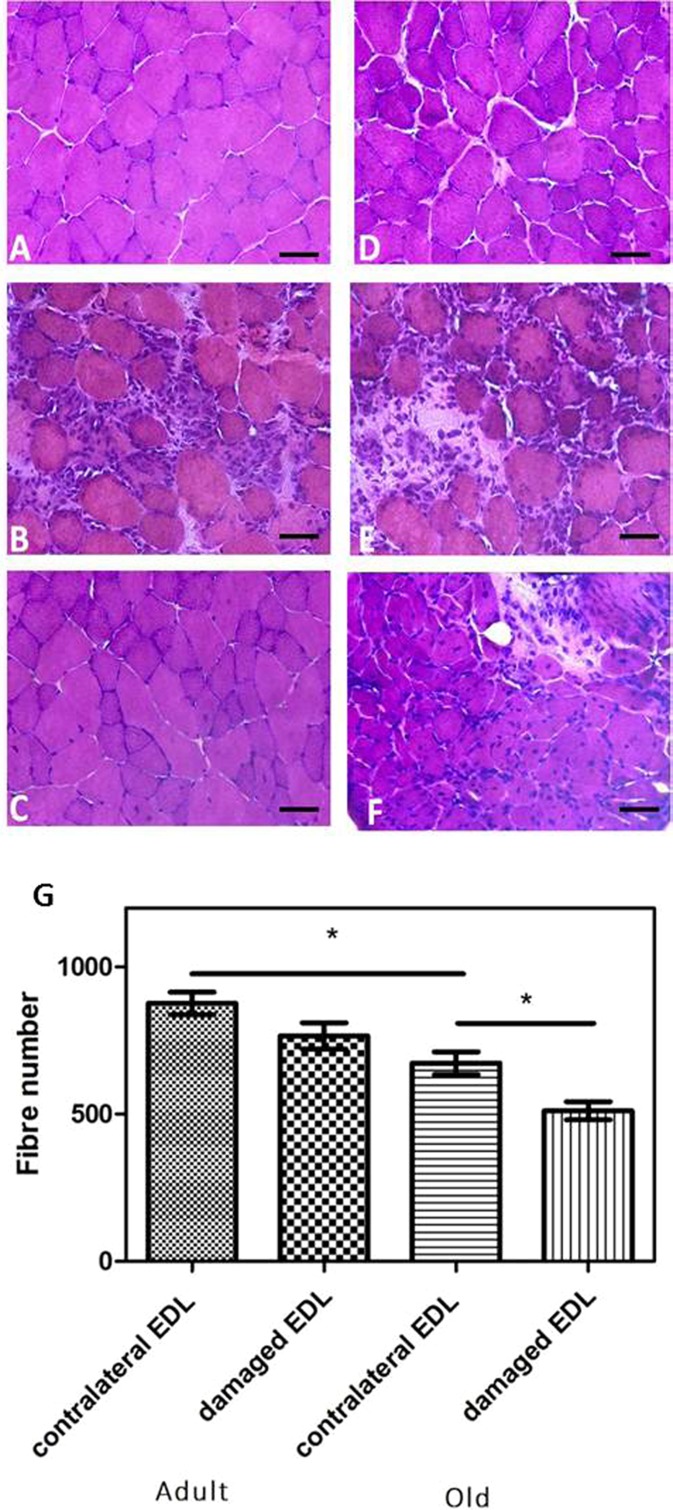
Representative transverse sections of quiescent EDL muscles from adult (**a**) and old (**d**) mice, EDL muscles 3 days post-lengthening contractions from adult (**b**) and old (**e**) mice and EDL muscles 28 days post-lengthening contractions from adult (**c**) and old (**f**) mice. Sections stained with hematoxylin and eosin. *Scale bar* 25 μm. **g** Total number of fibres per cross-section of contralateral control and experimental EDL muscles at 28 days following contraction-induced injury. Data collected from four animals. Values presented as mean ± s.e.m. *P < 0.05

The time course of changes in structure of the EDL muscles was assessed using histological approaches. Three days after contraction-induced injury, histological analysis of transverse sections of EDL muscles from adult mice showed widespread necrosis with the presence of phagocytic cells within fibres (Fig. 2b) compared with EDL muscles from the contralateral control leg (Fig. 2a). Twenty-eight days after lengthening contractions, transverse sections of muscles from adult mice appeared relatively normal with evidence of relatively few fibres with central nuclei present (Fig. 2c).

Quiescent muscle fibres of old mice (Fig. 2d) showed some minor changes in structure compared with those from adult mice (Fig. 2a). These included some variation in fibre size and an apparent increase in the extracellular space between fibres. At 3 days following the damaging lengthening contractions, muscle fibres from old mice showed evidence of substantial damage with infiltration of mononuclear cells (Fig. 2e) in a similar manner to muscles of adult mice (Fig. 2b). By 28 days post-contractions and in contrast to the pattern seen in muscles from adult mice, muscles from old mice showed multiple fibres with central nuclei, some residual infiltrating mononuclear cells and the presence of a large number of small fibres indicating that regeneration may be incomplete at that time point (Fig. 2f). Cross-sections from the control contralateral EDL muscles of old mice showed a reduced fibre number compared with the control EDL muscles of adult mice (673 ± 38 vs 876 ± 39; Fig. 2g). Lengthening contractions resulted in no significant change in the fibre number of EDL muscles of adult mice at 28 days following damage compared with undamaged muscles of the contralateral leg (766 ± 45 and 876 ± 39, respectively; Fig. 2g). In contrast, at 28 days after lengthening contractions, cross-sections of muscles of old mice showed a significant reduction in fibre number (512 ± 31) when compared with cross-sections from the contralateral leg (673 ± 38; Fig. 2g).

**Fig. 3:**
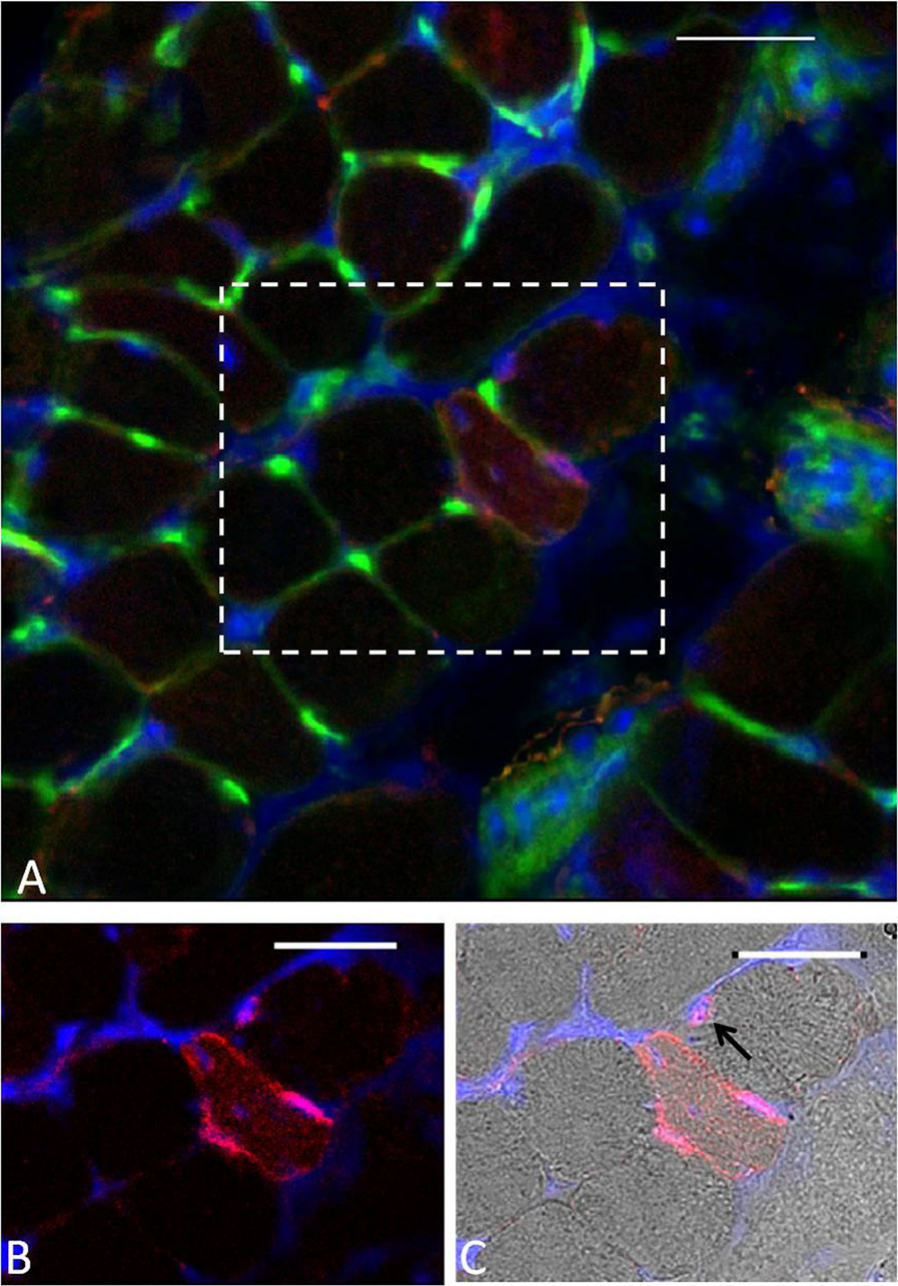
**a** Example of WGA–fluorescein lectin staining (*green*) and NCAM immunostaining (*red*) on a transverse cross-section of an EDL muscle from an old mouse at 3 days following damaging lengthening contractions. DAPI staining (*blue*) was used to visualise the nuclei. **b** Focused image of the denervated fibre. **c** Bright field image of the denervated fibre. *Black arrow* indicates satellite cell. *Scale bar* 30 μm

Positive immunostaining for NCAM is commonly used as a marker of denervated fibres in histological studies, and NCAM immunostaining was undertaken on transverse sections of EDL muscles from adult and old mice at 3 and 28 days post-contractions. Only a small number of NCAM-positive muscle fibres were seen in EDL muscles from both adult and old mice at any time point (<2 % per cross-section) with no differences between muscles of quiescent adult and old mice or within or between age groups at any time following damage (data not shown). An example of an NCAM-positive fibre is shown in Fig. 3a, c. As previously suggested by other authors, muscle sections stained with this anti-NCAM antibody (Kostrominova 2010; Kostrominova et al. 2005) also showed some NCAM staining of satellite cells (arrow in Fig. 3c).

### Innervation and structure of NMJs in EDL muscles from adult mice following lengthening contractions

Transgenic mice that express YFP in all motor axons (Thy1-YFP mice; (Feng et al. 2000)) were used to allow the ready location and examination of peripheral motor axons and pre-synaptic terminals of NMJs. Acetylcholine receptors (AChRs) in the postsynaptic muscle membrane were also labelled with fluorescently tagged α-bungarotoxin.

**Fig. 4:**
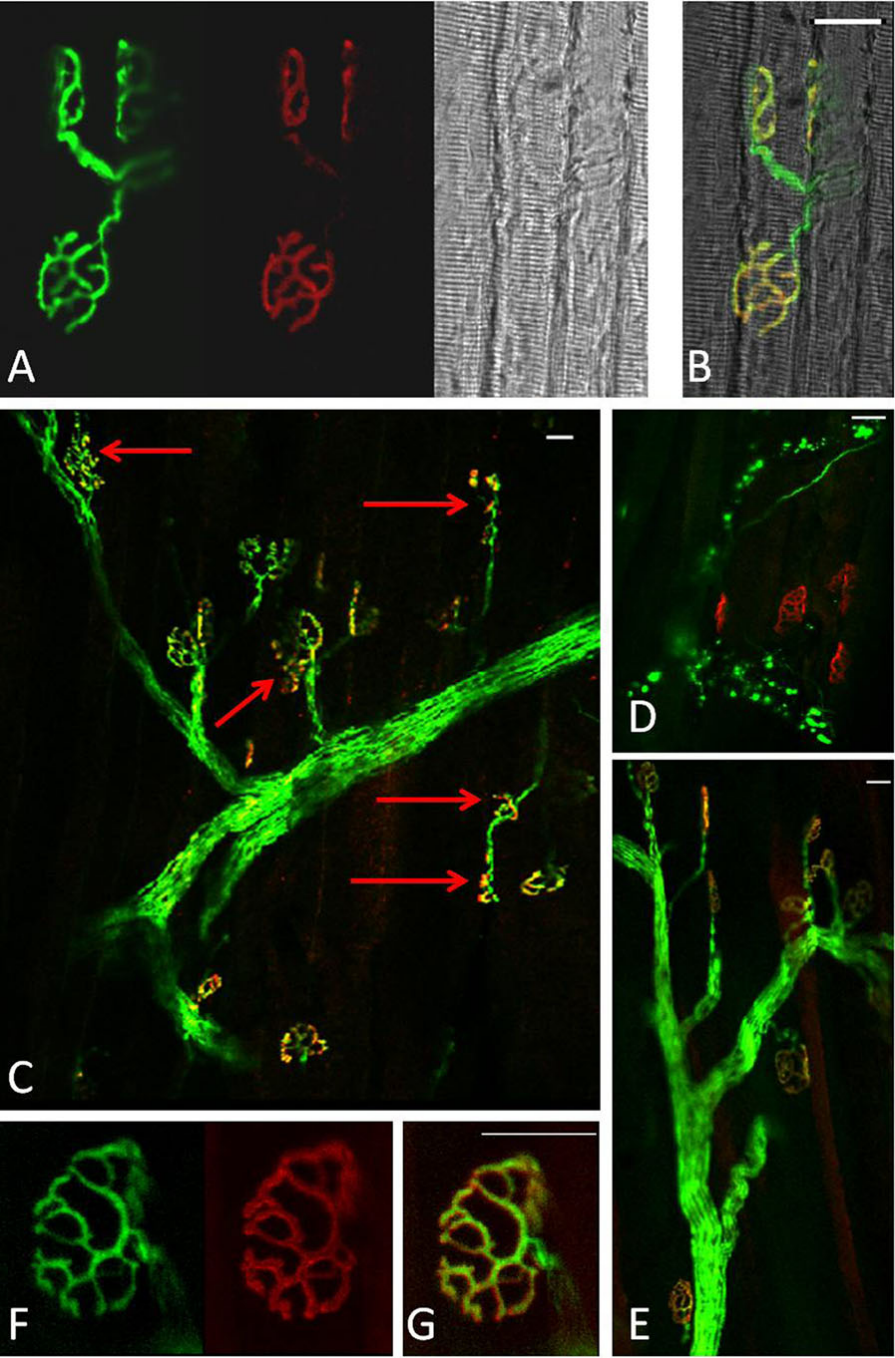
**a** Representative longitudinal section of a quiescent EDL muscle from an adult mouse showing YFP in motor axons (*green*), AChRs labeled with Alexa-594-α-bungorotoxin (*red*) and image of the fibres under bright field. **b** Merged image from **a. c–d** Representative longitudinal sections of EDL muscles from adult mice at 3 days following damage and **e** at 28 days post-damage. **f–g** Higher magnification of an NMJ of an EDL muscle at 28 days following damage. *Red arrows* indicate fragmented and denervated NMJs. *Scale bar* 30 μm

In quiescent muscles of adult mice, >99 % of the postsynaptic motor endplates and presynaptic nerve terminals observed were intact, well organised and compact with AChRs aggregates forming continuous long branches, each precisely aligned with an axonal branch (Figs. 4a, b and 8a, b). Three days following damage, approximately 60 % of the NMJs from muscles of adult mice showed substantial structural fragmentation. Example images are shown in Fig. 4c with quantification of the changes in Fig. 8a. Approximately 15 % of NMJs appeared to lack contact with an intact axon and hence were fully denervated at this time point (Figs. 4d and 8b). At 28 days following damage, approximately 96 % of the NMJs in EDL muscles of adult mice had restored normal structure (Figs. 4e and 8a) with less than 2 % of the total NMJs appearing to remain fully denervated (Fig. 8b) and without any other major structural abnormalities evident at 3 days following damage (Fig. 4e, f, g).

**Fig. 5:**
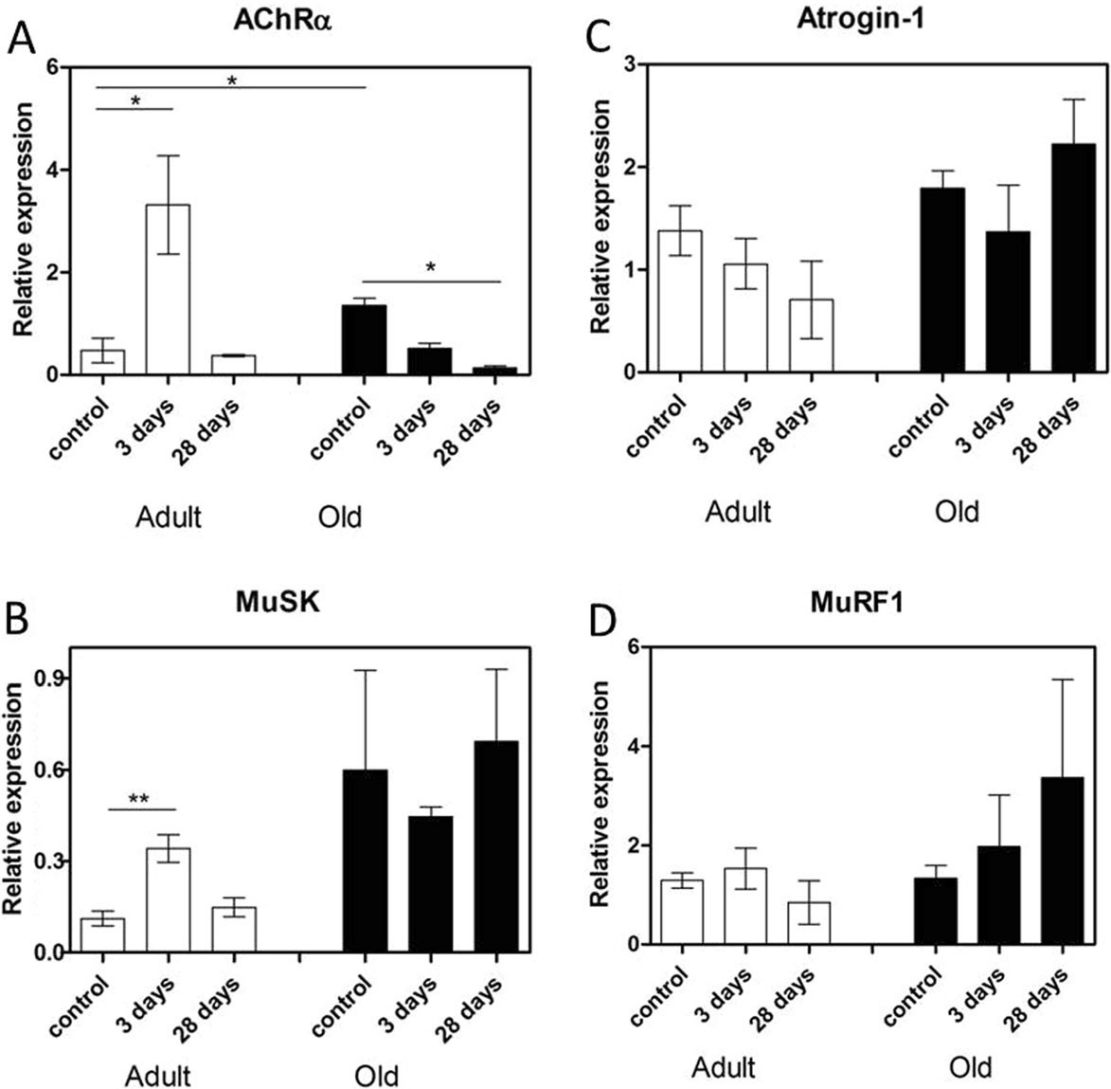
mRNA levels of denervation and atrophy markers. **a** AChRα, **b** MuSK, **c** Atrogin-1 and **d** MuRF1 mRNA expression in quiescent EDL muscles from adult and old mice, muscles at 3 days post-lengthening contractions and EDL muscles at 28 days post-lengthening contractions. Values presented as mean ± s.e.m

To determine whether lengthening contractions resulted in changes in markers of denervation, changes in expression of AChRα and MuSK (proteins essential to the formation and maintenance of NMJs ((Wang et al. 2006); (Punga et al. 2011)) were determined. The transcripts for these genes were increased significantly in the EDL muscles from adult mice at 3 days following damage and had returned to basal levels by 28 days (Fig. 5a, b).

To determine whether lengthening contractions resulted in changes in markers of atrophy, the expression of Atrogin-1 and MuRF1 (shown to be upregulated in many models of atrophy (Bodine et al. 2001)) were also determined. No changes were observed in the expression of these genes in muscles of adult or old mice either at rest or following contraction-induced damage (Fig. 5c, d).

### Innervation and structure of NMJs in EDL muscles of old mice following lengthening contractions

**Fig. 6:**
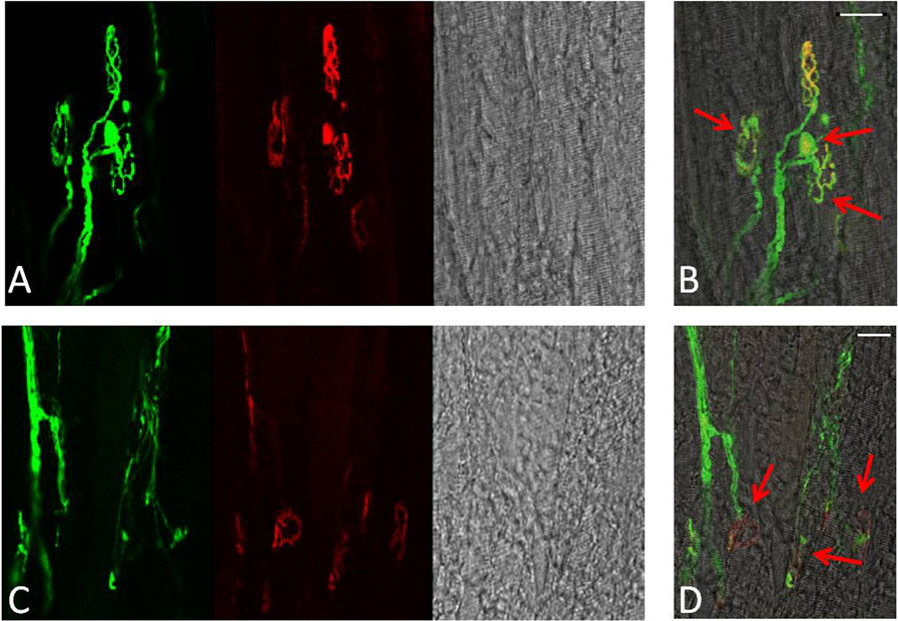
Representative longitudinal sections of EDL muscles from quiescent old mice showing YFP in motor axons (*green*), AChRs labelled with Alexa-594-α-bungorotoxin (*red*) and image of the fibres under bright field (**a**, **c**). **b**, **d** Merged image. *Red arrows* show age-related structural changes including nerve terminal fragmentation with some nerve terminals forming spherical and partially or fully denervated NMJs. *Scale bar* 30 μm

In contrast to muscles of adult mice, NMJs in quiescent EDL muscles of old mice showed a variety of structural alterations, including nerve terminal fragmentation with the majority of nerve terminals showing spherical ends as well as motor endplate disorganisation (Fig. 6a, b, c). These changes were seen in approximately 80 % of the NMJs observed. In addition, approximately 15 % of the NMJs in quiescent EDL muscles of old mice appeared to lack contact with an intact axon and so were fully denervated (Fig. 6c, d).

**Fig. 7:**
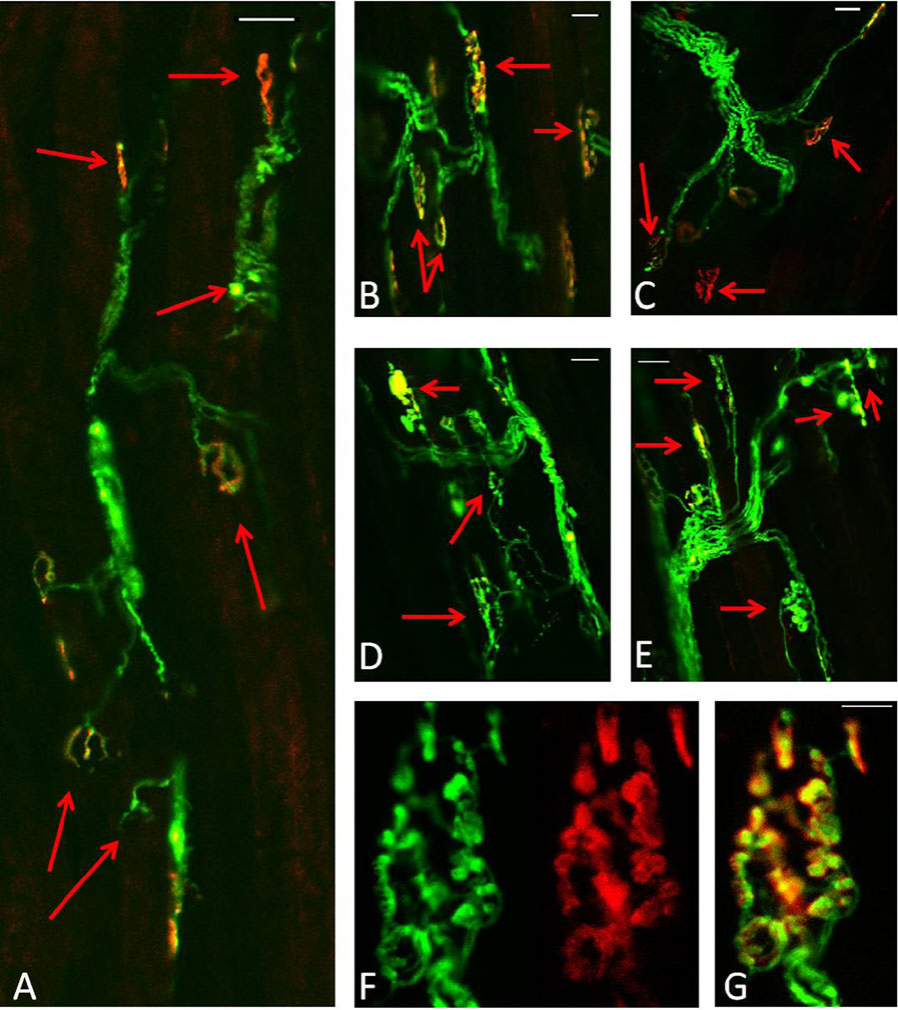
Longitudinal sections of EDL muscles from old mice expressing YFP in motor axons (*green*). **a** Three days following damage, **b–c** 28 days post damage, **d–e** 60 days following damage. AChRs labelled with Alexa-594-BTX (*red*). *Scale bar* 30 μm. **f–g** Higher magnification of a NMJ from EDL muscle of an old mouse 60 days following damage. AChRs labelled with Alexa-594-BTX (*red*). *Scale bar* 10 μm

**Fig. 8:**
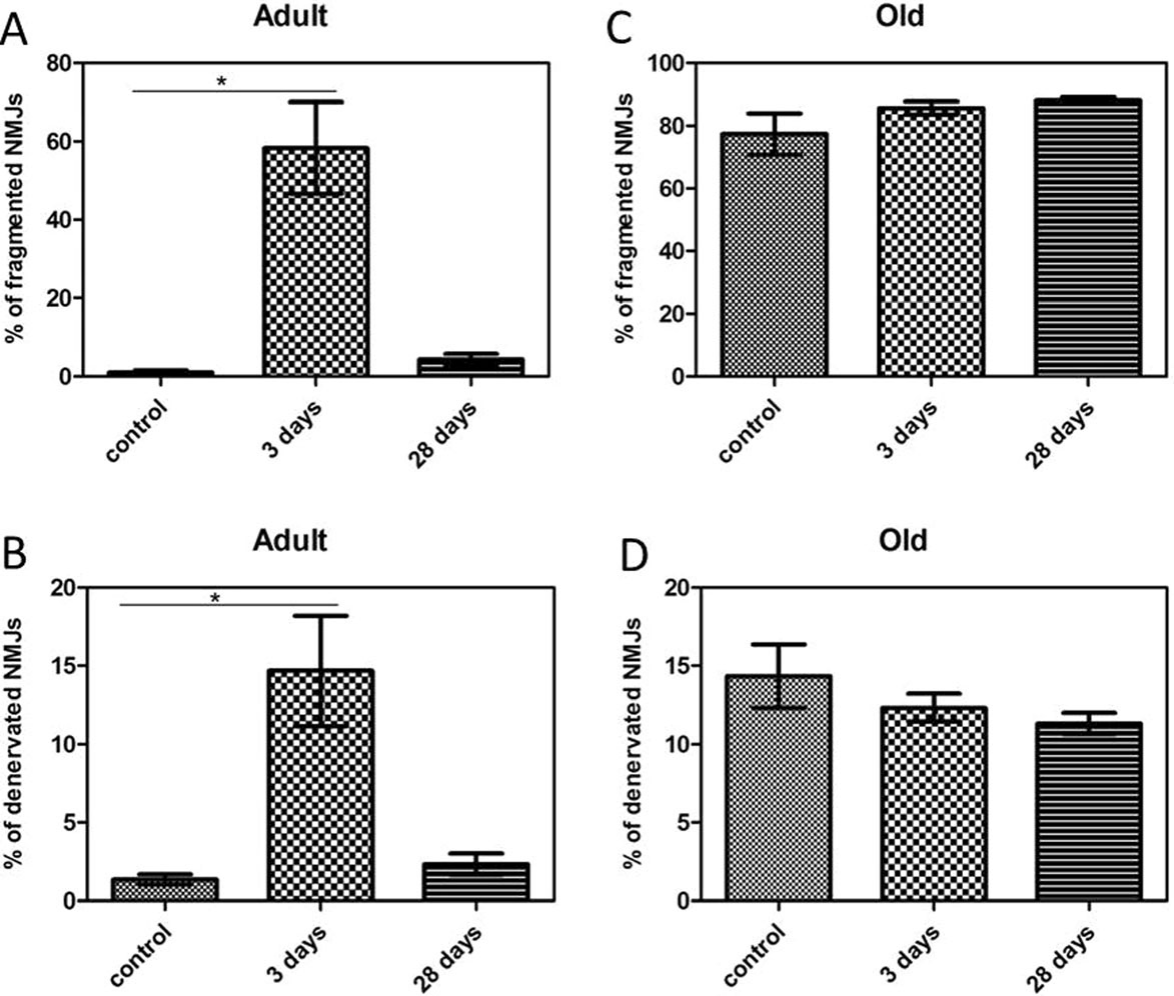
Percent of **a** fragmented and **b** fully denervated NMJs in EDL muscles from adult mice and percent of **c** fragmented and **d** fully denervated NMJs in EDL muscles from old mice at rest, 3 and 28 days following damaging lengthening contractions. Values presented as mean ± s.e.m. Data collected from 100 NMJs per EDL from 4 animals at each time point

Qualitatively, NMJs in muscles of old mice following damage showed greater disruption (Fig. 7a–c), but no significant changes were seen in the number of disrupted NMJs or fully denervated NMJs at 3 or 28 days compared with the contralateral undamaged EDL muscle (Fig. 8c, d).

In order to examine whether the permanent loss of force reported in muscles of old mice is associated with further structural alterations of the NMJs, a group of old mice were additionally allowed to recover for 60 days following the damaging protocol. Data show that at 60 days following damage, the majority of NMJs in EDL muscles from old mice remained disrupted, with severe structural alterations (Fig. 7f, g), showing granular fragmentation and swelling (Fig. 7d, e). Relatively few fully intact NMJs could be seen.

The expression of both AChRα and MuSK was significantly increased in quiescent EDL muscles of old mice compared with quiescent muscles of adult mice (Fig. 5a, b). However, the expression of Atrogin-1 and MuRF1 was not increased (Fig. 5c, d). The expression of AChRα was significantly decreased at 28 days following damage in EDL muscles of old mice compared with EDL muscles from quiescent old mice (Fig. 5a) whereas the expression of MuSK, Atrogin-1 and MuRF1 remained unchanged (Fig. 5b, c, d respectively).

## Discussion

The profound inability of muscles of old mice to regenerate fully following damage is potentially catastrophic for older individuals. This failure to re-innervate muscle during regeneration following damage is proposed to be a key mechanism in the development of sarcopenia although this hypothesis remains controversial. Despite this, little is known about any changes in motor neurons and neuromuscular junction (NMJ) structure following contraction-induced muscle damage in old mice.

The aim of this work was to examine the effect of a contraction-induced damage protocol on the structure of the peripheral motor neurons and NMJs in EDL muscles of adult and old mice. We hypothesised that (i) lengthening contraction-induced damage to muscles is associated with disruption of the innervation of muscle fibres that would be fully reversed during regeneration in adult mice but that (ii) the late stage failure in ability of muscles from old mice to recover their ability to generate force following lengthening contractions would be associated with a failure to appropriately re-innervate regenerated muscle fibres.

### Lengthening contractions induce defined levels of damage and regeneration in mice in vivo

A well-characterised protocol of lengthening contractions was used in the current study. EDL muscles from adult and old mice were subjected to lengthening contractions via the motor nerve as previously described (McArdle et al. 2004; McCully and Faulkner 1985). Damage occurring during this severe protocol of lengthening contractions follows a well-defined course of events in muscles of adult mice (Brooks and Faulkner 1990; Kayani et al. 2008; McArdle et al. 2004). Initially, the ability of muscle to generate force is reduced. At this stage, focal injury to single or groups of sarcomeres is observed. This initial injury leads to a secondary injury, classically at 3 days post-contractions, where widespread necrosis is evident within the muscle and at this point, a further reduction in the force production is observed. Data from the current study have now shown that this is associated with a clear upregulation of AChRα expression in muscle of adult mice, suggesting that active remodelling is occurring, although no changes in markers of muscle atrophy were seen. The muscle then slowly regenerates and force generation recovers to pre-exercise values by ∼28 days following contractions in adult mice, but a substantial deficit in the ability to generate force remains in muscles of old mice at this time point (Brooks and Faulkner 1990; McArdle et al. 2004). This is associated with the persistence of muscle fibres with central nuclei and small cross-sectional areas at 28 days possibly reflecting the presence of poorly innervated muscle fibres. The deficit in maximum force generation in muscles of old mice at 28 days following damage (McArdle et al. 2004) appears to be permanent (Brooks and Faulkner 1990) and is associated with loss of ∼25 % of muscle fibres at the same time point. Since muscle fibres are lost and small atrophic fibres are observed routinely in skeletal muscles of old mice (Brooks and Faulkner 1988, 1990), these authors proposed that contraction-induced injury may contribute to the development of sarcopenia.

### Innervation and structure of NMJs in EDL muscles from adult mice following lengthening contractions

Data support the hypothesis that the protocol of lengthening contractions to muscles of adult mice resulted in disruption of NMJ structure which can be fully reversed by 28 days post-contractions. It is possible that a number of the visibly disrupted NMJs are still able to function to elicit force generation by muscle fibres and that other factors such as direct focal damage to muscle fibres contribute to the overall loss of muscle force generation, particularly at 3 days following damage. Indeed, studies in acetylcholinesterase knockout mice demonstrate a pattern of acetylcholinesterase receptors which are distributed in a smaller and fragmented surface area, mirroring the branching pattern of motor nerve terminals but such patterns still allow an effective neuromuscular transmission. These findings indicate that the neuromuscular system exhibits a remarkable plasticity that has important consequences for the functioning of the neuromuscular junction (Girard et al. 2005).

### Structure and innervation of NMJs in quiescent EDL muscles of old mice and following lengthening contractions

Comparison of NMJs from quiescent adult and old mice revealed a variety of age-related structural alterations, swellings, partial or complete withdrawal of axons from some postsynaptic motor endplates and fragmentation of the postsynaptic organization in NMJs from old mice. This was associated with an increased expression of AchRα, suggesting increased remodelling. Similar age-related structural alterations have been reported by other groups in quiescent EDL (Chai et al. 2011) and tibialis anterior muscles (Valdez et al. 2010) from old mice and in muscles from mouse models of accelerated muscle ageing (Jang et al. 2010). Other age-related morphological and functional changes within and around NMJs have been reported, including a reduction in both the number of post-synaptic folds and the nerve terminal area which results in functional impairment in the post-synaptic response of the NMJ (Kurokawa et al. 1999) as well as mitochondrial alterations in axon terminals characterised by swelling, mitochondrial fusion and the presence of megamitochondria (Garcia et al. 2013). Previous data have shown that the specific force generation (force/CSA) by EDL muscles from old mice was reduced by approximately 25 % compared with that of adult mice when the muscles are activated via the motor nerve (McArdle et al. 2004) and so a comparison with the number of disrupted NMJs observed in quiescent muscle of old mice in this study (∼80 %) indicates that many of the apparently disrupted NMJs observed must retain some functional capacity. It has been hypothesised that the capacity of motor neurons to fully re-innervate muscle fibres that are denervated or regenerating is progressively reduced with ageing (Gonzalez-Freire et al. 2014) and data by Li et al (2011) has proposed that morphological changes in NMJs of old rodents are explained by degeneration and regeneration of muscle fibre segments at the synapse (Li et al. 2011).

We attempted to confirm the extent of denervation of the muscles by immunostaining of transverse muscle sections for NCAM as has previously been used (Kostrominova 2010; Kostrominova et al. 2005). However, only 1–2 % of fibres showed positive staining even at 3 days post-contractions where 15 % of fibres showed evidence of full denervation. In addition, no NCAM-positive muscle fibres were detected in EDL muscles from either adult or old mice at 28 days following lengthening contractions where approximately 12 % of muscle fibres from old mice still show denervation. Thus, we conclude that analysis of NCAM immunostaining at a single time point may underestimate the number of denervated fibres following contraction-induced damage. The likely explanation for this is the transient expression of NCAM in denervated fibres following lengthening contraction-induced damage, as has been proposed by other authors (Muller-Felber et al. 1993). In addition, it has been demonstrated that although muscle fibres of old mice retain normal background levels of NCAM, these muscles may have an impaired ability to upregulate extra-junctional NCAM in response to loss of motor nerve input (Gillon and Sheard 2015).

We hypothesised that degeneration of the muscle fibres induced by lengthening contractions would lead to further deterioration of NMJ structure in old mice. However, the pre-existence of such NMJ abnormalities in muscles of old mice made it difficult to determine the effect of lengthening contractions on NMJ disruption and recovery. Indeed, no further quantitative changes occurred at 3 or 28 days post-lengthening contractions despite substantial damage to the muscle fibres at 3 days following contraction-induced damage and a permanent substantial deficit in nerve-evoked force generation at 28 days following damage compared to pre-damage force generation. We hypothesised that the functional deficit in old mice at 28 days following contraction-induced damage would be associated with absent or incomplete re-innervation of the regenerating muscle fibres but no evidence for this was found. Surprisingly, axons of old mice navigate to and reoccupy former neuromuscular junctions following damage in a relatively efficient manner albeit with continued evidence of some NMJ abnormalities as seen in quiescent muscles. This is in agreement with the study of Kang and Lichtman (2013) that demonstrated that axons in old mice reoccupy neuromuscular junction sites more slowly, but efficiently although the authors state that only one of 42 NMJs examined in their study showed abnormalities including faint and fuzzy receptor staining, and in this instance, the overlay was not as efficient (Kang and Lichtman 2013). In order to fully understand the alack of effect of the contraction-induced damage on NMJ disruption in muscles of old mice, it is important to consider the nature of the approach used for assessing the percentage of disrupted NMJs and the changes in total muscle fibre number. The percentage of disrupted NMJs does not take into account the complete loss of fibres but reflects the percentage evident per 100 NMJs in each muscle. Fibre number remained the same in muscles of adult mice. However, in both the comparison of old control muscles with adult control muscles or in old muscles post-damage compared with old control muscles, the loss of muscle fibres and the impact of that on overall muscle function needs to be taken into consideration. It is not possible in the current study to identify the nature of the NMJs of those fibres that were completely lost, but given the lack of any statistical difference between the percentage of fragmented or fully denervated NMJs remaining in muscles of old mice at 28 days post-contractions compared with controls, it could be assumed that muscle fibres with either fragmented or completely denervated NMJs are lost. It is also clear from the previous discussion that many of the apparently disrupted NMJs observed in quiescent muscle from old mice at rest must retain some functional capacity and hence it is feasible that the contraction-protocol may have induced subtle changes in NMJ structure and function that were not detectable in the current study but that have a major effect on the ability of muscles to generate force following damaging contractions. Indeed, Verdu et al (1995) demonstrated that nerve regeneration following resection, judged by the reappearance of compound muscle action potential evoked by nerve stimulation amplitude, reached up to 90 % of control values in young mice following resection, but less than 50 % in old mice (Verdu et al. 1995). Our future studies will focus on determining neuromuscular transmission in parallel with determination of axonal and NMJ structure in order to evaluate such potential changes following contraction-induced injury.

### Nerve damage and disruption of NMJs are necessary to elicit the permanent functional deficit to muscles of old mice

Studies focussing on the ability of muscles of old rodents to regenerate following experimental damage by treatment with myotoxins or following nerve crush or resection or following grafting of muscles across different aged rodents has been very informative regarding the role of innervation in successful regeneration (Carlson et al. 2001; Kawabuchi et al. 2011; Lee et al. 2013; Streppel et al. 1998; Verdu et al. 1995). Myotoxins frequently damage muscle fibres, leaving the vasculature and nerve intact, compared with models which cause overt nerve damage (Grounds 2014). Transplantation results in spontaneous muscle degeneration and regeneration in the presence of a severed nerve and provides crucial information on the role of environmental factors on failed regeneration in muscles of old rodents (Brooks and Faulkner 1990). Based on these studies, the same group has proposed that the permanent deficit in muscle force generation following damage in old rodents requires peripheral nerve damage (Carlson and Faulkner 1996, 1998), supporting the hypothesis that lengthening contraction-induced muscle damage must contain a component of motor axon damage and the failure to regenerate successfully in this situation may be primarily due to the effects of changes in the local environment on re-innervation.

### Relevance of failed regeneration following contraction-induced damage to the development of sarcopenia

Previous studies from our laboratory in a transgenic mouse model of HSP70 overexpression has provided evidence that where the ability to regenerate muscles is preserved in old mice, this had little effect on the development of sarcopenia (McArdle et al. 2004), although the effect of overexpression of HSP70 on nerve structure and function in quiescent muscles or following the contraction protocol was not determined. Conversely, when muscle tetanic force generation and CSA are preserved by overexpression of HSP10, there was little effect on the development of the force deficit in muscles of old mice following a period of damaging lengthening contractions (Kayani et al. 2010). The study of Fry et al (2015) suggests that the ability of muscles to regenerate plays little role in the development of sarcopenia but data presented in this study and by others would suggest that in order to facilitate an accelerated development of sarcopenia, a compromised nerve regeneration process may also need to be present, a hypothesis that has also recently been proposed by Sataranatarajan and colleagues (Sataranatarajan et al. 2015).

In summary, our current and previously published data indicate that damage to muscle fibres induced by lengthening contractions in adult mice was associated with a 60–70 % loss of force production and damage to fibres that was further associated with an equivalent degree of disruption to NMJ structure and that the damage to the muscle fibres, neurons and NMJs was reversed entirely within 28 days. In contrast, in old mice, a substantial proportion of fibres (∼15 %) were denervated and NMJs disrupted (∼80 %) prior to contractions and no change in values for quantitative structural changes to NMJs were evident following the contraction-induced damage. Thus, data suggests that the disrupted NMJs evident in muscles of old mice must retain some functional capacity and that in old mice, the failure of regeneration that occurs following experimental damage does not appear to be associated with a specific failure of re-innervation of muscle fibres. In contrast, the complete loss of muscle fibres in old mice, at least in part, provides an explanation for the inability of muscles of old mice to recover the force deficit evident following contraction-induced damage and provides evidence that contraction-induced damage may play a role in the loss of muscle fibres in sarcopenia, albeit over a longer period of time.

## Abbreviations

EDL: Extensor digitorum longus
NMJs: Neuromuscular junctions
AChRs: Acetylcholine receptors
BTX: Bungarotoxin
NCAM: Neural cell adhesion molecule
WGA: Wheat germ agglutinin

## References

[CR1] Bodine SC et al. (2001) Identification of ubiquitin ligases required for skeletal muscle atrophy. Science (New York, N Y) 294:1704-1708 10.1126/science.1065874 11679633

[CR2] Brooks SV, Faulkner JA (1988). Contractile properties of skeletal muscles from young, adult and aged mice. J Physiol.

[CR3] Brooks SV, Faulkner JA (1990). Contraction-induced injury: recovery of skeletal muscles in young and old mice. Am J Physiol.

[CR4] Brooks SV, Zerba E, Faulkner JA (1995). Injury to muscle fibres after single stretches of passive and maximally stimulated muscles in mice. J Physiol.

[CR5] Brown WF, Strong MJ, Snow R (1988). Methods for estimating numbers of motor units in biceps-brachialis muscles and losses of motor units with aging. Muscle Nerve.

[CR6] Campbell MJ, McComas AJ, Petito F (1973). Physiological changes in ageing muscles. J Neurol Neurosurg Psychiatry.

[CR7] Carlson BM, Dedkov EI, Borisov AB, Faulkner JA (2001). Skeletal muscle regeneration in very old rats. J Gerontol A Biol Sci Med Sci.

[CR8] Carlson BM, Faulkner JA (1996). The regeneration of noninnervated muscle grafts and marcaine-treated muscles in young and old rats. J Gerontol A Biol Sci Med Sci.

[CR9] Carlson BM, Faulkner JA (1998). Muscle regeneration in young and old rats: effects of motor nerve transection with and without marcaine treatment. J Gerontol A Biol Sci Med Sci.

[CR10] Chai RJ, Vukovic J, Dunlop S, Grounds MD, Shavlakadze T (2011). Striking denervation of neuromuscular junctions without lumbar motoneuron loss in geriatric mouse muscle. PLoS One.

[CR11] Delbono O (2003). Neural control of aging skeletal muscle. Aging Cell.

[CR12] Demontis F, Piccirillo R, Goldberg AL, Perrimon N (2013). The influence of skeletal muscle on systemic aging and lifespan. Aging Cell.

[CR13] Ehrhardt J, Morgan J (2005). Regenerative capacity of skeletal muscle. Curr Opin Neurol.

[CR14] Einsiedel LJ, Luff AR (1992). Alterations in the contractile properties of motor units within the ageing rat medial gastrocnemius. J Neurol Sci.

[CR15] Faulkner JA, Brooks SV, Opiteck JA (1993). Injury to skeletal muscle fibers during contractions: conditions of occurrence and prevention. Phys Ther.

[CR16] Faulkner JA, Brooks SV, Zerba E (1995) Muscle atrophy and weakness with aging: contraction-induced injury as an underlying mechanism. The Journals of Gerontology Series A, Biological Sciences and Medical Sciences 50 Spec No:124-129 10.1093/gerona/50a.special_issue.124 7493205

[CR17] Faulkner JA, Jones DA, Round JM (1989). Injury to skeletal muscles of mice by forced lengthening during contractions. Q J Exp Physiol.

[CR18] Feng G (2000). Imaging neuronal subsets in transgenic mice expressing multiple spectral variants of GFP. Neuron.

[CR19] Friden J, Sjostrom M, Ekblom B (1983). Myofibrillar damage following intense eccentric exercise in man. Int J Sports Med.

[CR20] Fry CS (2015). Inducible depletion of satellite cells in adult, sedentary mice impairs muscle regenerative capacity without affecting sarcopenia. Nat Med.

[CR21] Garcia ML, Fernandez A, Solas MT (2013). Mitochondria, motor neurons and aging. J Neurol Sci.

[CR22] Gillon A, Sheard P (2015). Elderly mouse skeletal muscle fibres have a diminished capacity to upregulate NCAM production in response to denervation. Biogerontology.

[CR23] Girard E, Barbier J, Chatonnet A, Krejci E, Molgo J (2005). Synaptic remodeling at the skeletal neuromuscular junction of acetylcholinesterase knockout mice and its physiological relevance. Chem Biol Interact.

[CR24] Goljanek-Whysall K, Mok GF, Fahad Alrefaei A, Kennerley N, Wheeler GN, Munsterberg A (2014). myomiR-dependent switching of BAF60 variant incorporation into Brg1 chromatin remodeling complexes during embryo myogenesis. Development.

[CR25] Gonzalez-Freire M, de Cabo R, Studenski SA, Ferrucci L (2014). The neuromuscular junction: aging at the crossroad between nerves and muscle frontiers in aging. Neuroscience.

[CR26] Gonzalez E, Delbono O (2001). Age-dependent fatigue in single intact fast- and slow fibers from mouse EDL and soleus skeletal muscles. Mech Ageing Dev.

[CR27] Grounds MD (2014). The need to more precisely define aspects of skeletal muscle regeneration. Int J Biochem Cell Biol.

[CR28] Jang YC (2010). Increased superoxide in vivo accelerates age-associated muscle atrophy through mitochondrial dysfunction and neuromuscular junction degeneration. FASEB J.

[CR29] Jang YC, Van Remmen H (2011). Age-associated alterations of the neuromuscular junction. Exp Gerontol.

[CR30] Kang H, Lichtman JW (2013). Motor axon regeneration and muscle reinnervation in young adult and aged animals. J Neurosci.

[CR31] Kawabuchi M, Tan H, Wang S (2011). Age affects reciprocal cellular interactions in neuromuscular synapses following peripheral nerve injury. Ageing Res Rev.

[CR32] Kayani AC, Close GL, Broome CS, Jackson MJ, McArdle A (2008). Enhanced recovery from contraction-induced damage in skeletal muscles of old mice following treatment with the heat shock protein inducer 17-(allylamino)-17-demethoxygeldanamycin. Rejuvenation Res.

[CR33] Kayani AC, Close GL, Dillmann WH, Mestril R, Jackson MJ, McArdle A (2010). Overexpression of HSP10 in skeletal muscle of transgenic mice prevents the age-related fall in maximum tetanic force generation and muscle Cross-Sectional Area. Am J Physiol Regul Integr Comp Physiol.

[CR34] Kostrominova TY (2010). Advanced age-related denervation and fiber-type grouping in skeletal muscle of SOD1 knockout mice. Free Radic Biol Med.

[CR35] Kostrominova TY (2011). Application of WGA lectin staining for visualization of the connective tissue in skeletal muscle, bone, and ligament/tendon studies. Microsc Res Tech.

[CR36] Kostrominova TY, Dow DE, Dennis RG, Miller RA, Faulkner JA (2005). Comparison of gene expression of 2-mo denervated, 2-mo stimulated-denervated, and control rat skeletal muscles. Physiol Genomics.

[CR37] Kurokawa K, Mimori Y, Tanaka E, Kohriyama T, Nakamura S (1999) Age-related change in peripheral nerve conduction: compound muscle action potential duration and dispersion . Gerontology 45:168-173 doi:22081. 10.1159/000022081 10202263

[CR38] Larsson L, Ansved T (1995). Effects of ageing on the motor unit. Prog Neurobiol.

[CR39] Lee AS (2013). Aged skeletal muscle retains the ability to fully regenerate functional architecture. Bioarchitecture.

[CR40] Lexell J, Downham D, Sjostrom M (1986). Distribution of different fibre types in human skeletal muscles. Fibre type arrangement in m. vastus lateralis from three groups of healthy men between 15 and 83 years. J Neurol Sci.

[CR41] Lexell J, Taylor CC, Sjostrom M (1988). What is the cause of the ageing atrophy? Total number, size and proportion of different fiber types studied in whole vastus lateralis muscle from 15- to 83-year-old men. J Neurol Sci.

[CR42] Li Y, Lee Y, Thompson WJ (2011). Changes in aging mouse neuromuscular junctions are explained by degeneration and regeneration of muscle fiber segments at the synapse. J Neurosci.

[CR43] McArdle A, Dillmann WH, Mestril R, Faulkner JA, Jackson MJ (2004). Overexpression of HSP70 in mouse skeletal muscle protects against muscle damage and age-related muscle dysfunction. FASEB J.

[CR44] McCully KK, Faulkner JA (1985). Injury to skeletal muscle fibers of mice following lengthening contractions. J Appl Physiol.

[CR45] McCully KK, Faulkner JA (1986). Characteristics of lengthening contractions associated with injury to skeletal muscle fibers. J Appl Physiol.

[CR46] Miller RA (2004). ‘Accelerated aging’: a primrose path to insight?. Aging Cell.

[CR47] Muller-Felber W (1993). Fibre type specific expression of Leu19-antigen and N-CAM in skeletal muscle in various stages after experimental denervation. Virchows Arch A Pathol Anat Histopathol.

[CR48] Porter MM, Vandervoort AA, Lexell J (1995). Aging of human muscle: structure, function and adaptability. Scand J Med Sci Sports.

[CR49] Punga AR, Maj M, Lin S, Meinen S, Ruegg MA (2011). MuSK levels differ between adult skeletal muscles and influence postsynaptic plasticity. Eur J Neurosci.

[CR50] Rader EP, Faulkner JA (2006). Effect of aging on the recovery following contraction-induced injury in muscles of female mice. J Appl Physiol.

[CR51] Sataranatarajan K (2015). Neuron specific reduction in CuZnSOD is not sufficient to initiate a full sarcopenia phenotype. Redox Biol.

[CR52] Shi X, Garry DJ (2006). Muscle stem cells in development, regeneration, and disease. Genes Dev.

[CR53] Soriano-Arroquia A, McCormick R, Molloy AP, McArdle A, Goljanek-Whysall K (2016). Age-related changes in miR-143-3p:Igfbp5 interactions affect muscle regeneration. Aging Cell.

[CR54] Streppel M, Angelov DN, Guntinas-Lichius O, Hilgers RD, Rosenblatt JD, Stennert E, Neiss WF (1998). Slow axonal regrowth but extreme hyperinnervation of target muscle after suture of the facial nerve in aged rats. Neurobiol Aging.

[CR55] Valdez G, Tapia JC, Kang H, Clemenson GD, Gage FH, Lichtman JW, Sanes JR (2010). Attenuation of age-related changes in mouse neuromuscular synapses by caloric restriction and exercise. Proc Natl Acad Sci U S A.

[CR56] Verdu E, Buti M, Navarro X (1995). The effect of aging on efferent nerve fibers regeneration in mice. Brain Res.

[CR57] Wang Q, Zhang B, Xiong W-C, Mei L (2006). MuSK signaling at the neuromuscular junction. J Mol Neurosci.

[CR58] Wang ZM, Zheng Z, Messi ML, Delbono O (2005). Extension and magnitude of denervation in skeletal muscle from ageing mice. J Physiol.

